# Complete plastome of *Leucanthemum maximum*, the first in genus *Leucanthemum*

**DOI:** 10.1080/23802359.2019.1693922

**Published:** 2019-12-09

**Authors:** Haimei Chen, Mei Jiang, Liqiang Wang, Jinwen You, Chang Liu

**Affiliations:** a Key Laboratory of Bioactive Substances and Resource Utilization of Chinese Herbal Medicine from Ministry of Education, Engineering Research Center of Chinese Medicine Resources from Ministry of Education, Institute of Medicinal Plant Development, Chinese Academy of Medical Sciences and Peking Union Medical College, Beijing, P. R. China;; b Institute of Chinese Herbal Medicine, Hubei Academy of Agricultural Sciences, Enshi, Hubei, P. R. China

**Keywords:** *Leucanthemum maximum*, plastome, phylogenetic analysis

## Abstract

*Leucanthemum maximum* is a perennial herb widely used in landscaping. Here, we reported the complete plastome of *L. maximum*. The plastome was 151,865 bp long, containing a large single-copy region of 84,369 bp, a small single-copy region of 18,450 bp, and two inverted repeats of 24,523 bp each. It encoded 112 unique genes, including 79 protein-coding, 4 rRNA, and 29 tRNA genes. The protein sequence of *infA* was distinctly truncated compared with those from other Anthemideae species. Phylogenetic analysis showed that the species is closely related to the genus *Ismelia*. This study provided a high-quality reference for future studies.

*Leucanthemum maximum* is a flowering plant from family Asteraceae, tribe Anthemideae. It is native to France and Spain, but it can be found to grow in the wild in other parts of the world as an introduced species and sometimes cultivated as an ornamental plant (Wikipedia 2019). *L. maximum* has a ploidy level of 2*n* = 12*x* = 108. The obtainment of the plastome is the first step to understand the phylogeny and phylogeography of a polyploidy complex (Greiner et al. 2012). For this reason, we sequenced and analyzed the complete plastome from *L. maximum* in this study.

The fresh leaves of *L. maximum,* identified by Professor Jinwen You, were collected from the Central China Medicinal Botanical Garden, EnShi, Hubei, China (Geospatial coordinate: N30.177764, E109.743937). The voucher sample is stored in the herbarium of Institute of Medicinal Plant Development (201808226). The genomic DNA was extracted with plant genomic DNA kit (Tiangen Biotech, China) and sequenced using the HiSeq 2500 platform (Illumina, San Diego, CA) following the manufacturer’s recommendations. The plastome was assembled with NOVOPlasty (v.2.7.2) (Dierckxsens et al. 2017) and annotated with CPGAVAS2 (Shi et al. 2019). The annotated genomic sequence has been submitted to GenBank with the accession number: MN518843.

The plastome of *L. maximum* is a typical circular DNA molecule with a total length of 151,865 bp. It has the conservative quadrilateral structure, including a large single-copy (LSC) region, a small single-copy (SSC) region, and a pair of inverted repeat (IR) regions, the length was 84,369, 18,450, and 24,523 bp. The overall GC content is 37.33%. The GC content of three regions is ranked as 43.10%, 35.41%, and 30.74% for IRs, LSC, and SSC, respectively. The plastome of *L. maximum* encoded 79 unique protein coding, 29 unique tRNA genes, and 4 unique rRNA genes. Among them, nine protein-coding genes (*rps*16, *rpo*C1, *atp*F, *pet*B, *pet*D, *rpl*16, *rpl*2, *ndh*A, and *ndh*B) contained one intron, and two protein-coding genes (*ycf*3 and *clp*P) contained two introns. The *rps*12 gene is a trans-spliced gene with the 5′ end located in the LSC region and the duplicated 3′ ends located in the IR regions. Four tRNA genes (*trn*K-UUU, *trn*S-CGA, *trn*E-UUC, and *trn*A-UGC) contained one intron. The *inf*A gene coded a protein of 41 residues, significantly shorter than those from other Anthemideae species.

For the phylogenetic analysis, 75 protein sequences present in 17 additional Anthemideae species, two outgroup species (*Nymphoides coreana* and *Menyanthes trifoliata*), and *L. maximum* were retrieved using the ‘DownloadCOG’ module in PLasDB (http://www.herbalgenomics.org/plasdb). These protein sequences were aligned using the CLUSTALW2 (v2.0.12) program. The maximum likelihood method implemented in RaxML (v8.2.4) (Stamatakis 2015) was used to infer the evolutionary history, using the model of PROTGAMMACPREV. Subsequently, the bootstrap analysis was performed with 1000 replicates. As shown in Figure 1, *L. maximum* is closely related to the genus of *Ismelia*, with bootstrap values of 96, consistent with the current taxonomic classification. In the future, more plastome sequences from other *Leucanthemum* species are needed to determine their phylogenetic relationships.

**Figure 1.**
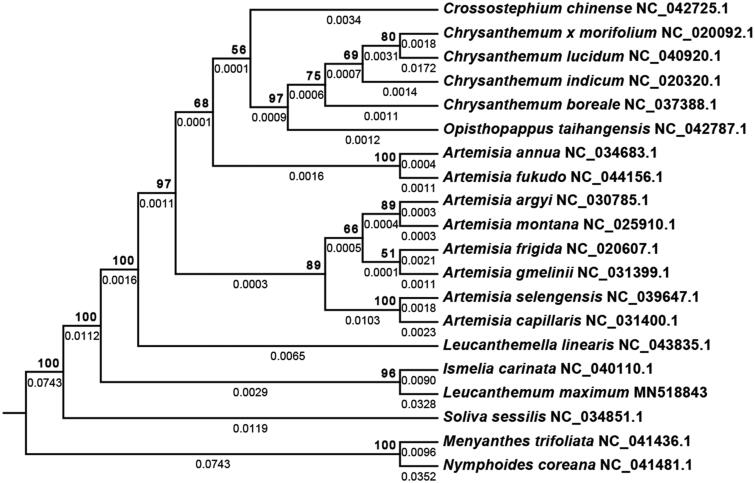
Molecular phylogenetic analyses of plastomes in the tribe Anthemideae. The tree was constructed with the sequences of 75 proteins present in all 20 species by using the maximum-likelihood method implemented in RAxML. *Nymphoides coreana* and *Menyanthes trifoliata* were set as outgroups. The number on the top of the branch indicates bootstrap value, and the number at the bottom of the branch indicates the evolutionary distance. The accession numbers for the species are as follows: *Artemisia annua*: NC_034683.1, *Artemisia argyi*: NC_030785.1, *Artemisia capillaris*: NC_031400.1, *Artemisia frigida*: NC_020607.1, *Artemisia fukudo*: NC_044156.1, *Artemisia gmelinii*: NC_031399.1, *Artemisia montana*: NC_025910.1, *Artemisia selengensis*: NC_039647.1, *Chrysanthemum boreale*: NC_037388.1, *Chrysanthemum lucidum*: NC_040920.1, *Crossostephium chinense*: NC_042725.1, *Ismelia carinata*: NC_040110.1, *Leucanthemella linearis*: NC_043835.1, *Opisthopappus taihangensis*: NC_042787.1, *Soliva sessilis*: NC_034851.1, *Chrysanthemum indicum*: NC_020320.1, *Chrysanthemum morifolium*: NC_020092.1, *Nymphoides coreana*: NC_041481.1, and *Menyanthes trifoliata*: NC_041436.1.
